# Activator- and repressor-type MYB transcription factors are involved in chilling injury induced flesh lignification in loquat via their interactions with the phenylpropanoid pathway

**DOI:** 10.1093/jxb/eru208

**Published:** 2014-05-24

**Authors:** Qian Xu, Xue-ren Yin, Jiao-ke Zeng, Hang Ge, Min Song, Chang-jie Xu, Xian Li, Ian B. Ferguson, Kun-song Chen

**Affiliations:** ^1^Laboratory of Fruit Quality Biology/The State Agriculture Ministry Laboratory of Horticultural Plant Growth, Development and Quality Improvement, Zhejiang University, Zijingang Campus, Hangzhou 310058, PR China; ^2^New Zealand Institute for Plant & Food Research Limited, Private Bag 92169, Auckland, New Zealand

**Keywords:** Chilling injury, flesh lignification, heat treatment, Loquat, low-temperature condition, MYB, transcriptional regulation.

## Abstract

Lignin biosynthesis and its transcriptional regulatory networks have been studied in model plants and woody trees. However, lignification also occurs in some fleshy fruit and has rarely been considered in this way. Loquat (*Eriobotrya japonica*) is one such convenient tissue for exploring the transcription factors involved in regulating fruit flesh lignification. Firmness and lignin content of ‘Luoyangqing’ loquat were fund to increase during low-temperature storage as a typical symptom of chilling injury, while heat treatment (HT) and low-temperature conditioning (LTC) effectively alleviated them. Two novel *EjMYB* genes, *EjMYB1* and *EjMYB2*, were isolated and were found to be localized in the nucleus. These genes responded differently to low temperature, with *EjMYB1* induced and *EjMYB2* inhibited at 0 °C. They also showed different temperature responses under HT and LTC conditions, and may be responsible for different regulation of flesh lignification at the transcriptional level. Transactivation assays indicated that *EjMYB1* and *EjMYB2* are a transcriptional activator and repressor, respectively. *EjMYB1* activated promoters of both *Arabidopsis* and loquat lignin biosynthesis genes, while *EjMYB2* countered the inductive effects of *EjMYB1*. This finding was also supported by transient overexpression in tobacco. Regulation of lignification by *EjMYB1* and *EjMYB2* is likely to be achieved via their competitive interaction with AC elements in the promoter region of lignin biosynthesis genes such as *Ej4CL1.*

## Summary

Two novel *MYB* transcription factors are involved in lignin biosynthesis and flesh lignification in loquat fruit, which are manipulated by temperature condition and treatments.

## Introduction

Lignin is important for plant secondary cell-wall formation, and lignin biosynthesis and the associated regulatory mechanisms have been studied in the model plant *Arabidopsis*, various crops, and woody trees (Goicoechea *et al.*, 2005; Zhong and Ye, 2009; Vanholme *et al.*, 2010; Zhao and Dixon, 2011). Monolignols of lignin are synthesized via the phenylpropanoid pathway and then oxidatively polymerized to lignin polymers. Genes encoding the enzyme cascade of the phenylpropanoid pathway have also been widely characterized. For instance, *Arabidopsis* mutants, such as *ref3* (cinnamate 4-hydroxylase, *C4H*) (Ruegger *et al.*, 1999), *ref8* (*p*-coumarate 3-hydroxylase, *C3H*) (Franke *et al.*, 2002*a*, *b*), and *irx4* (cinnamoyl-CoA reductase, *CCR*) (Jones *et al.*, 2001), have been shown to have significantly reduced lignin content. Antisense 4-coumarate:CoA ligase (*4CL*) transgenic *Arabidopsis* plants have also been shown to have reduced lignin contents as well as changes in lignin composition (Lee *et al.*, 1997).

*MYB* genes constitute a very large transcription factor family with diverse functions, involved in many biological processes including lignin biosynthesis (Dubos *et al.*, 2010). Based on mutants and genetic mapping, many lignin biosynthesis-related MYB transcription factors have been cloned and characterized in different plants. In *Arabidopsis*, more than nine *AtMYB*s have been reported, and they function either as activators or repressors. *AtMYB61* (Newman *et al.*, 2004), *AtMYB46* (Zhong *et al.*, 2007), *AtMYB85* (Zhong *et al.*, 2008), *AtMYB58* and *AtMYB63* (Zhou *et al.*, 2009), and *AtMYB83* (McCarthy *et al.*, 2009) have all been identified as activators, and *AtMYB4* (Jin *et al.*, 2000) and *AtMYB32* (Preston *et al.*, 2004) as repressors. Among the activators, only two members, *AtMYB58* and *AtMYB63*, have been identified as lignin specific (Zhou *et al.*, 2009), and so far no lignin-specific repressor has been reported in *Arabidopsis*. Most lignin-associated MYB members interact with the AC elements of promoters of target genes (Lacombe *et al.*, 2000; Zhou *et al.*, 2009).

In plants other than *Arabidopsis*, such as *Eucalyptus*, *Pinus taeda*, *Populus trichocarpa*, *Antirrhinum majus*, and *Zea mays*, lignin biosynthesis has also been found to be regulated by activator or repressor MYB family members. *EgMYB2* positively regulated lignin biosynthesis in stems when overexpressed in tobacco (Goicoechea *et al.*, 2005). *PtMYB1* and *PtMYB8* (Bomal *et al.*, 2008) and *PtrMYB3* and *PtrMYB20* (McCarthy *et al.*, 2010) have been reported as activators, and *AmMYB308*, *AmMYB330* (Tamagnone *et al.*, 1998), *EgMYB1* (Legay *et al.*, 2007), *ZmMYB42* (Sonbol *et al.*, 2009), and *ZmMYB31* (Fornalé *et al.*, 2010) as repressors of lignin biosynthesis. However, few studies have reported the interaction of activators and repressors in regulation of lignin biosynthesis. In addition, almost all the previous work on MYB-based regulation of lignin biosynthesis has focused on vegetative tissues/organs, whereas lignin biosynthesis can occur in other tissues such as fleshy fruit, which may provide good models that differ in phenolpropanoid synthesis and environmental responses.

Flesh lignification significantly influences fruit texture, which eventually affects storability and fruit quality (Li *et al.*, 2010). Loquat [*Eriobotrya japonica* Lindl. cv. ‘Luoyangqing’ (LYQ)] fruit are susceptible to chilling injury, exhibited as substantial flesh lignification during postharvest cold storage (Cai *et al.*, 2006*b*; Yang *et al.*, 2008; Wang *et al.*, 2010). The dynamics of lignin biosynthetic enzymes, e.g. phenylalanine ammonia lyase (PAL), 4CL, cinnamyl alcohol dehydrogenase (CAD), and peroxidase (POD), have been studied in association with lignin content in LYQ fruit (Cai *et al.*, 2006*a*; Shan *et al.*, 2008). Postharvest technologies such as low-temperature conditioning (LTC), where fruit are exposed to non-damaging low temperatures to induce a degree of low-temperature tolerance, and exposure to high temperatures, are effective in alleviating flesh lignification caused by chilling injury during long-term cold storage (Cai *et al.*, 2006*b*). This provides a biological system where gene response to temperatures that affect lignin synthesis can be investigated, providing greater insight into regulation and the effects of the external environment.

In the present study, two loquat *MYB* genes, *EjMYB1* and *EjMYB2*, were isolated based on homologue studies with *Arabidopsis* MYBs of known function, and were expected to be a transcriptional activator and repressor, respectively. Transcripts of the *EjMYB* genes in response to different temperature treatments, including heat treatment (HT) and LTC, were investigated with regard to the regulation of fruit lignification. Transcriptional regulatory mechanisms and putative function characterization and interaction of these two *EjMYB* genes were performed with dual luciferase and transient expression systems in tobacco leaves and a yeast one-hybrid system.

## Materials and methods

### Plant material and treatments

Commercially mature loquat LYQ (*Eriobotrya japonica* Lindl.) fruit were harvested from an orchard at Luqiao, Zhejiang province, China, in 2011. The fruit were transported to the laboratory on the day of harvest. Fruit of uniform maturity with a mean firmness of 4.14 N, and with absence of disease and mechanical wounding, were selected and divided into three batches, each comprising 150 fruit. The first batch was treated at 40 °C for 4h and then transferred to 0 °C storage (HT); the second batch was stored at 5 °C for 6 d and then transferred to 0 °C storage (LTC); the third batch was stored directly at 0 °C as the control.

Three replicates, each of five fruit, were sampled at each sampling time. The skins and stones were removed to provide three combined flesh samples. The tissues were frozen in liquid nitrogen and stored at –80 °C until further use.

### Fruit firmness

Fruit firmness is one of the main indices used to monitor postharvest lignification of loquat fruit (Cai *et al*., 2006*a*, *b*). Fruit firmness was measured using a TA-XT plus Texture Analyser (Stable Micro Systems, UK), with a 5mm diameter probe, a penetration rate of 1mm s^–1^ and a penetration depth of 4mm (Shan *et al.*, 2008; Wang *et al.*, 2010). The firmness of each fruit was averaged from two measurements 90° apart at the fruit equator, after removal of a small piece of peel. Fruit firmness was expressed as newtons (N) and 10 individual fruit replicates were used.

### Lignin content determination

Lignin content determination was according the methods described by Shan *et al.* (2008) and Wang *et al.* (2010). The frozen sample was ground into a powder and homogenized in 5ml of washing buffer (100mM K_2_HPO_4_/KH_2_PO_4_, 0.5% Triton X-100, 0.5% PVP, pH 7.8). The mixture was cultured on a shaker at room temperature at 200rpm for 30min, and then centrifuged (6000*g*, 25 °C) for 20min. The pellet was suspended and washed twice in washing buffer as above and then four times in 100% methanol. The pellet was dried at 80 °C in an oven overnight. Ten milligrams of the dry power was dissolved in 1.0ml of 2.0M HCl and 0.1ml of thioglycolic acid. The mixture was then boiled in a water bath (100 °C) for 8h, and then cooled on ice for 5min before centrifugation at 10 000*g* for 20min at 4 °C. The pellet was washed with distilled water and suspended in 2.0ml of 1.0M NaOH. After agitating lightly at room temperature for 18h, the solution was centrifuged at 10 000*g* for 20min. Supernatant (0.5ml) was transferred to a new tube with 0.1ml of concentrated HCl. The tubes were left at 4 °C for 4h to precipitate the lignin thioglycolic acid, followed by centrifugation at 10 000*g* for 20min at 4 °C, and the precipitate was dissolved in 1ml of 1.0M NaOH. Absorbance was measured at 280nm, using 1.0M NaOH as the blank. Data were expressed on a fresh weight basis, and all measurements were done in triplicate.

### Gene/promoter isolation and analysis

Two *EjMYB* genes were isolated based on the known lignin-specific *Arabidopsis AtMYB58*, *AtMYB63* (Zhou *et al.*, 2009) and *AtMYB4* (Jin *et al.*, 2000) genes. Oligonucleotide primers were designed, using the apple genome for reference sequences (Supplementary Table S1 at *JXB* online). The untranslated region of two *EjMYB* sequences was amplified using a SMART RACE cDNA Amplification kit (Clontech) and the primers listed in Supplementary Table S2 at *JXB* online.

Promoters of lignin biosynthesis-related genes were prepared from both *Arabidopsis* and loquat. Promoters of the *Arabidopsis* lignin biosynthesis genes were isolated according to the sequences in The *Arabidopsis* Information Resource (TAIR), using the primers shown in Supplementary Table S3 at *JXB* online.

Loquat lignin biosynthesis genes *EjPAL1* (EF685344), *Ej4CL1* (EF685345), *EjCAD1* (EF685346), and *EjCAD2* (EF685347) were isolated in our previous work (Shan *et al.*, 2008) and *Ej4CL2* (KF767455), *Ej4CL3* (KF767456), *Ej4CL4* (KF767457), *Ej4CL15* (KF767458), and *EjCAD3* (KF767459) were isolated using RNA sequencing (RNA-seq). Promoters of these genes were isolated with a GenomeWalker kit (Clontech), using the primers described in Supplementary Table S4 and the sequences are shown in Supplementary Fig. S2 at *JXB* online. *Cis*-elements (AC elements) in the promoters were analysed with both online software (http://bioinformatics.psb.ugent.be/webtools/plantcare/html/) (Lescot *et al.*, 2002) and the sequences indicated in Fornalé *et al.* (2010) [ACC(T/A)ACC].

Due to the length and numbers of AC elements in the promoter region, only the *Ej4CL1* promoter was chosen for further deletion experiments to test the interaction between *EjMYB* and AC elements, using the primers described in Supplementary Table S5 at *JXB* online.

Alignment was performed using the neighbour-joining method in ClustalX (v.1.81), and a phylogenetic tree was reconstructed with the online software Figtree (http://tree.bio.ed.ac.uk/software/figtree/). The deduced amino acid sequences of *Arabidopsis* MYB were obtained from TAIR.

### Subcellular localization analysis

The coding sequences of *EjMYB1* and *EjMYB2* were each cloned as C-terminal fusions in frame with the green fluorescent protein (GFP) gene into the pBI221 vector and expressed under the control of the cauliflower mosaic virus (CaMV) 35S promoter (primers are listed in Supplementary Table S6 at *JXB* online). The fusion constructs 35S::EjMYB1-GFP and 35S::EjMYB2-GFP and control vector pBI221 (35S::GFP) were transfected with mCherry, which has a nuclear localization signal (NLS), into protoplasts obtained from suspension-cultured BY-2 tobacco (*Nicotiana tabacum*). The protoplasts were transfected using a modified polyethylene glycol method as described by Abel and Theologis (1994) and Wang *et al.* (2005), and were incubated for 16h at 25 °C. The transfected cells were analysed by fluorescence microscope (Zeiss). All transient expression assays were repeated at least three times.

### RNA extraction and cDNA synthesis

Total RNA was extracted from loquat flesh, according to the protocol described by Shan *et al.* (2008). Contaminating genomic DNA in the total RNA was removed a using TURBO DNA-free kit (Ambion). The total RNA was then quantified using Nanophotometer Pearl (Implen). RNA (3 μg) was used for cDNA synthesis with a Revert Aid^TM^ First Strand cDNA Synthesis kit (Fermentas). Tenfold-diluted cDNA was used for real-time PCR.

### Real-time PCR analysis

For real-time PCR, gene-specific oligonucleotide primers were designed and are described in Supplementary Table S7 at *JXB* online. The gene specificity of each pair of primers was double checked by melting curves and product resequencing, according to the procedures described by Yin *et al.* (2008). The *EjACT* gene was employed as the internal control for monitoring the abundance of the mRNA. The sequences of *EjACT* primers are described in Supplementary Table S7.

Real-time PCR were performed on a LightCycler 1.5 instrument (Roche), initiated by 5 min at 95 °C and followed by 45 cycles of 95 °C for 5 s, 60 °C for 5 s, and 72 °C for 10 s, and completed with a melting- curve analysis program. The PCR mixture (10 μl total volume) comprised 2 μl of 5× LightCycler FastStart DNA Master^PLUS^ SYBR Green I Master Mix (Roche), 0.5 μl of each primer (10 μM), 1 μl of diluted cDNA and 6μl PCR-grade H_2_O. No-template controls and melting-curve analysis were included for each gene during each run.

### Dual luciferase assay

Dual luciferase assays were performed according to our previous reports (Yin *et al.*, 2010; Min *et al.*, 2012). The promoters of the *Arabidopsis* and loquat lignin biosynthesis genes were amplified with the primers described in Supplementary Tables S3 and S4, respectively. Full-length *EjMYB1* and *EjMYB2* were inserted into the pGreen II 0029 62-SK vector (SK), while the promoters were inserted into the pGreen II 0800-LUC vector. Details of vector information are given in Hellens *et al.* (2005).

All the constructs were electroporated into *Agrobacterium tumefaciens* GV3101. The dual luciferase assays were performed with *Nicotiana benthamiana* leaves. *Agrobacterium* cultures were prepared with infiltration buffer (10mM MES, 10mM MgCl_2_, 150mM acetosyringone, pH 5.6) to an OD_600_ of 0.7–1.0. *Agrobacterium* culture mixtures of transcription factors (1ml) and promoters (100 µl) were infiltrated into tobacco leaves using needleless syringes. The tobacco plants were grown in a glasshouse with daylight extension to 16h. Three days after infiltration, firefly luciferase and *Renilla* luciferase were assayed using dual luciferase assay reagents (Promega). For each transcription factor–promoter interaction, three independent experiments were performed (at least five replicates in each experiments).

### Yeast one-hybrid assay

In order to verify the results obtained from the dual luciferase assay, yeast one-hybrid assays were performed using the Matchmaker Gold Yeast One-Hybrid Library Screening System (Clontech, USA). The promoter of *Ej4CL1* was constructed into pAbAi vector (the primers are listed in Supplementary Table S8 at *JXB* online). Ej4CL1–AbAi and p53–AbAi were linearized and transformed into Y1HGold. The full-length transcription factors *EjMYB1* and *EjMYB2* were subcloned into pGADT7 AD vector (the primers are listed in Supplementary Table S6). All of the constructs were transformed into Y1HGold[Ej4CL1/AbAi], separately, and then were cultured on SD/–Leu medium containing 0–200ng ml^–1^ of aureobasidin A (AbA) at 28 °C for 3 d to test for interaction. pGADT7-Rec was co-transformed with the p53 fragment into Y1HGold[p53/AbAi] and Y1HGold[Ej4CL1/AbAi] as positive and negative controls, respectively.

### Transient expression

In order to determine the roles of *EjMYB* genes in the regulation of lignin biosynthesis, an unstable transient expression transformation system was adapted. The transient expression analyses were performed in *N. tabacum*, using the same batch of *Agrobacterium* stock and infiltration buffer of the dual luciferase assay, according to the protocols described by Espley *et al.* (2007). Target genes (*MYB*) and negative controls (SK) were included on two sides of the same leaves. Five days after infiltration, the infiltrated leaves were sampled and used for lignin analysis. Tissue (0.5g) from each of the infiltrated leaves was taken for lignin analysis, making eight single leaf replicates in total. The transient expression analyses were repeated in at least three independent experiments.

Transient activation of the endogenous target genes *Nt4CL1* (U50845) and *Nt4CL2* (U50846) in tobacco were chosen to be analysed. The primers are described in Supplementary Table S7, and primers for *Nt4CL1* were used according to Omer *et al.* (2013).

### Statistical analysis

The statistical significance of differences was calculated using Student’s *t*-test. Least significant difference (LSD) at the 5% level was calculated using DPS7.05 (Zhejiang University, Hangzhou, China). Figures were drawn using Origin 8.0 (Microcal Software Inc., Northampton, MA, USA).

## Results

### *EjMYB* isolation and analysis

**Fig. 1.**
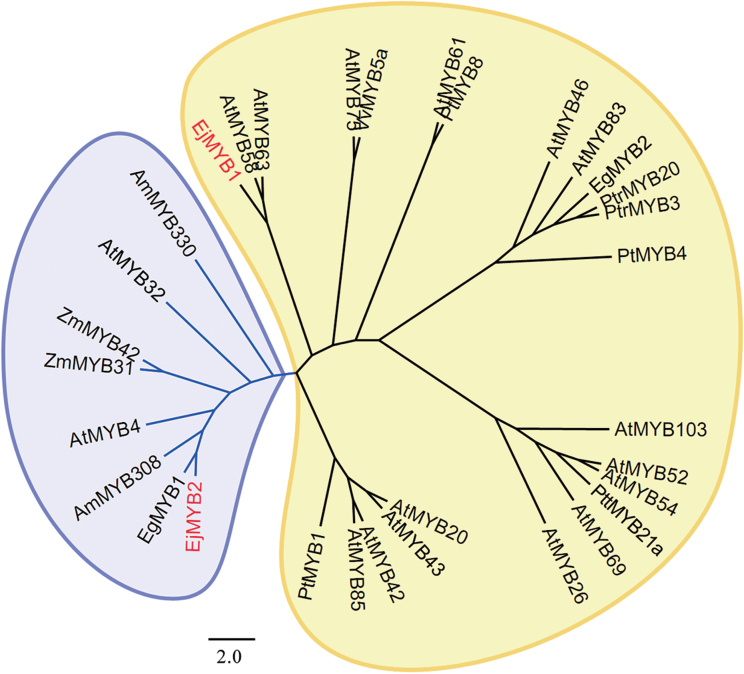
Phylogenetic analysis of loquat and other MYBs involved in the regulation of lignin biosynthesis or secondary wall metabolism. Blue lines represent transcriptional repressors. AtMYB4, AAS10085; AtMYB20, AT1G66230.1; AtMYB26, AT3G13890.1; AtMYB32, AT4G34990.1; AtMYB42,AT4G12350.1; AtMYB43, AT5G16600.1; AtMYB46, AT5G12870.1; AtMYB52, AT1G17950.1; AtMYB54, AT1G73410.1; AtMYB58, AT1G16490.1; AtMYB61, AT1G09540.1; AtMYB63, AT1G79180.1; AtMYB69, AT4G33450.1; AtMYB75, AT1G56650.1; AtMYB83, AT3G08500.1; AtMYB85, AT4G22680.1; AtMYB103, AT1G63910.1; PtMYB1, AY356372; PtMYB4, AY356371; PtMYB8, DQ399057.1; EgMYB1, CAE09058; EgMYB2, AJ576023; PtrMYB3, XM_002299908; PtrMYB20, XM_002313267; AmMYB308, P81393; AmMYB330, P81395; ZmMYB31, CAJ42202; ZmMYB42, CAJ42204; PttMYB21a, AJ567345; VvMYB5a, AY555190.

**Fig. 2.**
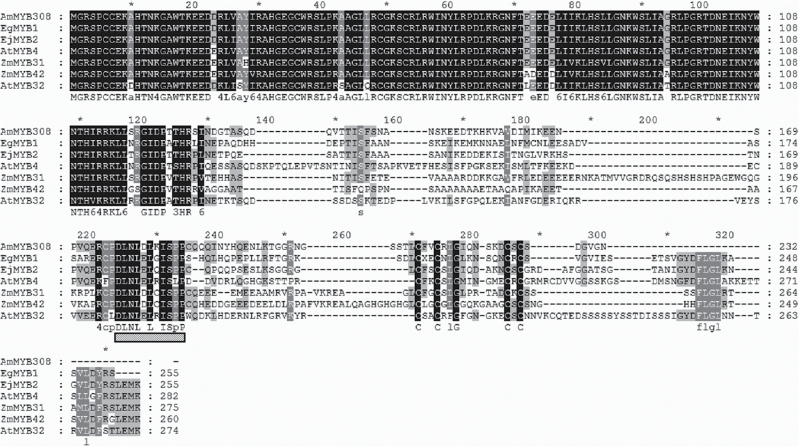
Alignment analysis of EjMYB2 and other repressor-type MYBs. The location of an EAR repressor domain is indicated with a gridlines bar. Black shading indicates 100% consensus amino acid sequence among the different genes, while the other colours represent lower levels of consensus.

Two *EjMYB* genes, designated *EjMYB1* (KF767453) and *EjMYB2* (KF767454), were isolated from loquat flesh. Based on the phylogenetic tree, EjMYB1 belongs to an activator-type MYB group and had high amino acid identity with AtMYB58 and AtMYB63, which are lignin-specific activators, while EjMYB2 belongs to a repressor-type group and clustered with EgMYB1, AmMYB308, AtMYB4 and others (Fig. 1). Alignment analysis indicated that EjMYB2 and other repressor-type MYBs have a conserved motif, named an ERF-associated amphiphilic repression domain (EAR) (Legay *et al.*, 2007), which is characteristic for repressor-type transcription factors (Fig. 2).

**Fig. 3.**
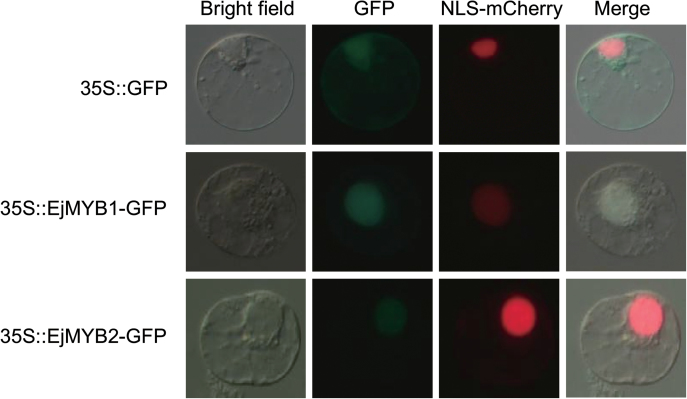
Subcellular localization analysis of EjMYB1 and EjMYB2. NLS–mCherry, which has nuclear localization, was co-transfected with 35S::GFP (empty vector), 35S:: EjMYB1*-*GFP, or 35S:: EjMYB2*-*GFP.

The full-length coding sequences of *EjMYB1* and *EjMYB2* without stop codes were constructed in pBI221. mCherry, which localizes to the nucleus, was co-transfected with 35S::EjMYB1–GFP, 35S::EjMYB2–GFP, and empty vector pBI221 (35S::GFP). The protoplasts with 35S::GFP showed green fluorescence throughout the cell and red fluorescence in the nucleus, while the protoplasts with 35S::EjMYB1–GFP or 35S::EjMYB2–GFP displayed both green and red fluorescence in the nucleus (Fig. 3).

### Expression responses of *EjMYB* genes in flesh of LYQ loquat fruit after temperature treatments

**Fig. 4.**
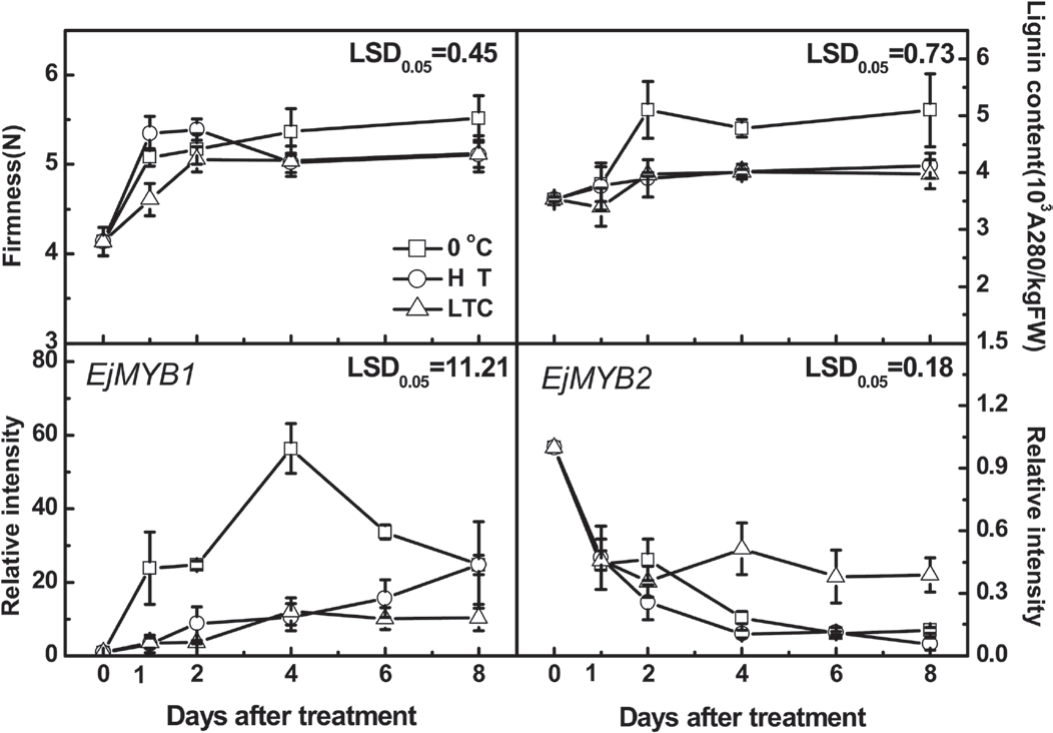
Effects of HT and LTC treatment on LYQ fruit firmness, lignin content, and expression of *EjMYB* genes. Gene expression was expressed as a ratio relative to the harvest time point (0 d), which was set as 1. Error bars indicate standard error (SE) from ten (firmness) or three (lignin and gene expression) replicates. LSDs represent least significant difference at 0.05.

The firmness of LYQ loquat fruit increased gradually during storage at 0 °C, with the firmness changing from 4.14 N at harvest to 5.17 N at 2 d and 5.52 N at 8 d (Fig. 4). After 4 d, the changes in firmness were consistent with changes in lignin content, where a 44% increase was observed (from 3.53×10^3^ to 5.10×10^3^ *A*_280_ kg^–1^ of fresh weight) at 0 °C for 8 d (Fig. 4). Both HT and LTC overall inhibited the increase in firmness and lignin content (Fig. 4). The inhibitory effects of the treatments were not observable until after 2 d, and after 8 d of storage, firmness was 5.11 and 5.12 N for HT and LTC-treated fruit, respectively. In parallel, the lignin content in fruit after 8 d of storage was 4.12×10^3^ *A*_280_ kg^–1^ of fresh weight for HT fruit and 3.98×10^3^ *A*_280_ kg^–1^ of fresh weight for LTC-treated fruit (Fig. 4).

Transcript analysis indicated that *EjMYB1* and *EjMYB2* responded differently to low temperature (Fig. 4). *EjMYB1* expression was rapidly induced at 0 °C, and its expression transiently increased and reached a maximum by 4 d. *EjMYB2* was repressed by low temperature, with levels continuing to decline over the 8 d. Both high-temperature and the LTC treatment inhibited the low-temperature stimulation of *EjMYB1*. Heat had little effect on the low-temperature induced decline in *EjMYB2* levels, while expression levels of *EjMYB2* were higher in the LTC-treated fruit than in control fruit after 4 d of storage at 0 °C (Fig. 4). There were general consistencies between increased or inhibited lignification and expression of the putative activator *EjMYB1*, but the slightly increased levels in response to heat were not reflected in the lignification pattern. In addition, apart from the general decline in the putative repressor *EjMYB2* levels, which were consistent with lignification, the responses to heat were not so consistent, although a simple model of maintaining repressor gene levels associated with reduced lignification did hold with the LTC response of this gene.

### *In vivo* interaction of *EjMYB* and promoters of lignin biosynthesis genes from *Arabidopsis*

**Fig. 5.**
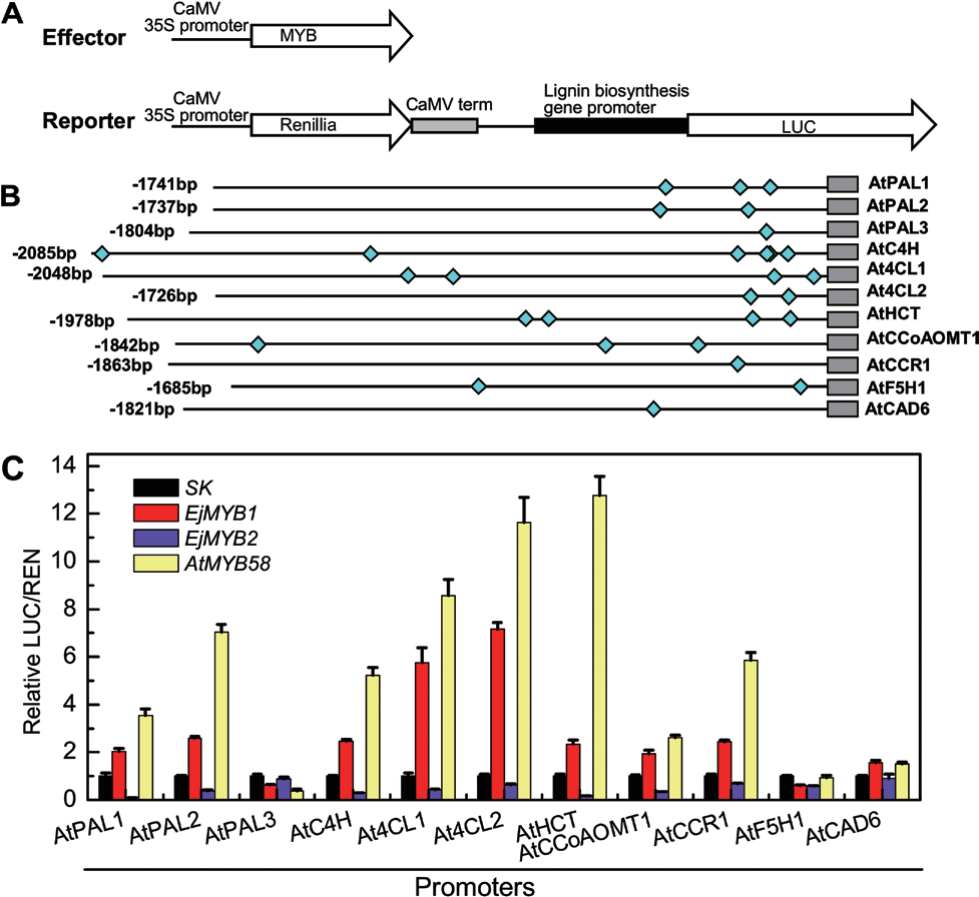
*In vivo* interaction of *EjMYB1*, *EjMYB2*, and *AtMYB58* with promoters of lignin biosynthesis genes from *Arabidopsis*. (A) Diagrams of the effector and reporter constructs used for the dual luciferase assay. (B) Schematics of promoters are indicated with lines (promoter length) and diamonds (AC elements). (C) *In vivo* associations of transcription factors and promoters obtained from transient assays in tobacco leaves. The ratio of LUC/REN of the empty vector (SK) plus promoter was used as a calibrator (set as 1). Error bars indicate SE from at least five replicates.

In order to investigate the transcriptional regulatory linkage between *EjMYB* genes and lignin biosynthesis, promoters of 11 functionally characterized genes in the phenylpropanoid pathway were isolated from *Arabidopsis* (Fig. 5B). These promoters ranged from 1685 to 2085bp and were rich in AC elements (Fig. 5B). A dual luciferase assay indicated that *EjMYB1*, which is similar to *AtMYB58*, activated most of the promoters, including *AtPAL1*, *AtPAL2*, *AtC4H*, *At4CL1*, *At4CL2*, *AtHCT*, *AtCCoAOMT1*, and *AtCCR1*, with a pattern across the genes very similar to that of the *Arabidopsis* MYB, while *EjMYB2* did not activate these promoters (Fig. 5A, C).

### *In vivo* interaction of *EjMYB* and promoters of *Ej4CL* genes from loquat

**Fig. 6.**
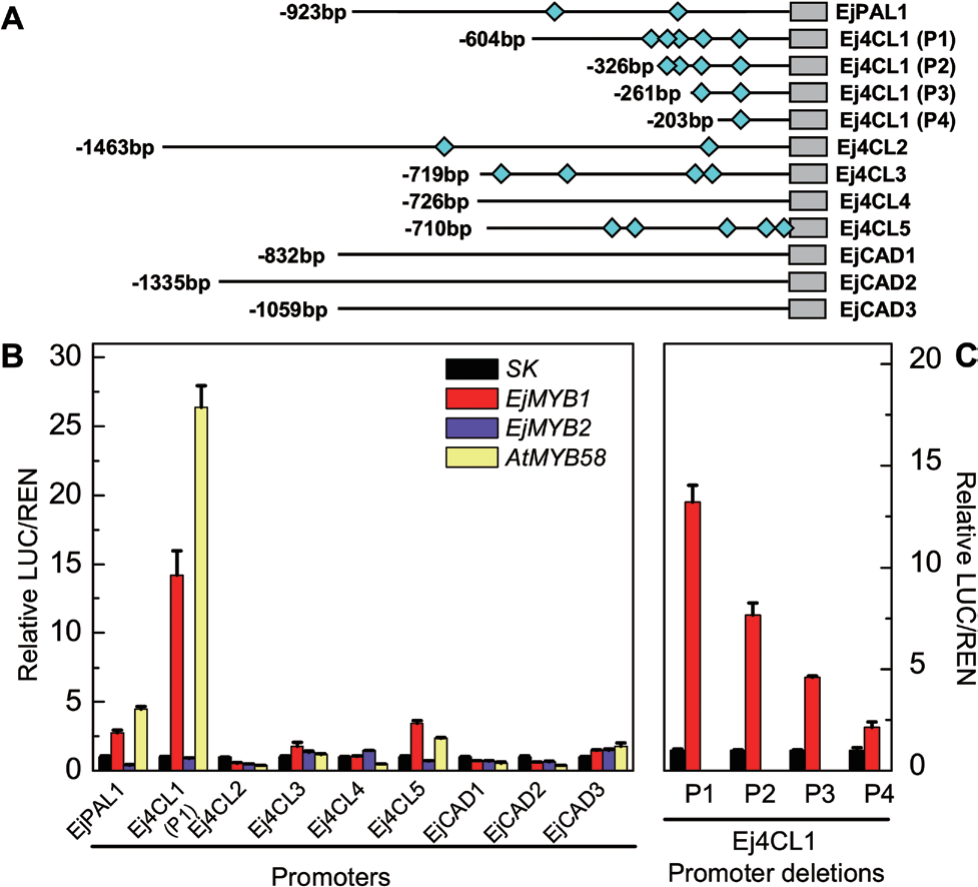
*In vivo* interaction of *EjMYB1*, *EjMYB2*, and *AtMYB58* with promoters of the *EjPAL*, *Ej4CL*, and *EjCAD* genes from LYQ fruit. (A) Schematics of the promoters are indicated with lines (promoter length) and diamonds (AC elements). (B, C) *In vivo* associations of transcription factors and promoters obtained from transient assays in tobacco leaves. The ratio of LUC/REN of the empty vector (SK) plus promoter was used as calibrator (set as 1). Error bars indicate SE from at least five replicates.

Due to the lack of genome information for loquat, mature LYQ fruit were analysed by RNA sequencing and unigenes associated with the lignin biosynthesis phenylpropanoid pathway were isolated. Within these gene families, eight genes were successfully isolated with full or partial-length coding sequences and promoters, including one *EjPAL*, five *Ej4CL* genes, and three *EjCAD* genes. Except for *Ej4CL4* and the *EjCAD* genes, at least one AC element was distributed in the promoter regions of the genes (Fig. 6A).

In the presence of *EjMYB1* or *AtMYB58*, the activities of the promoter of *Ej4CL1* were significantly induced, with some increase for *EjPAL1* and *Ej4CL5*, and the others showing no particular response beyond that of SK. The stimulatory effects of *EjMYB1* on the promoters of *EjPAL1*, *Ej4CL1*, and *Ej4CL5*, which are lignin related, reached ~3-, ~14- and ~3-fold, respectively (Fig. 6B). *Ej4CL1* and *Ej4CL5* showed similar phylogenetic patterns to those of class I (Supplementary Fig. S1 at *JXB* online), which has been associated with the monolignol biosynthesis pathway. To confirm that *EjMYB1* regulates lignin biosynthesis genes via binding to AC elements, further analysis was performed with the promoter of *Ej4CL1*, which had been induced ~14-fold and had five AC elements. The transactivation of *EjMYB1* on the *Ej4CL1* promoter was reduced by each deletion, accompanied by decreasing AC elements, while the fifth AC element (P4) was the least important (Fig. 6C).

**Fig. 7.**
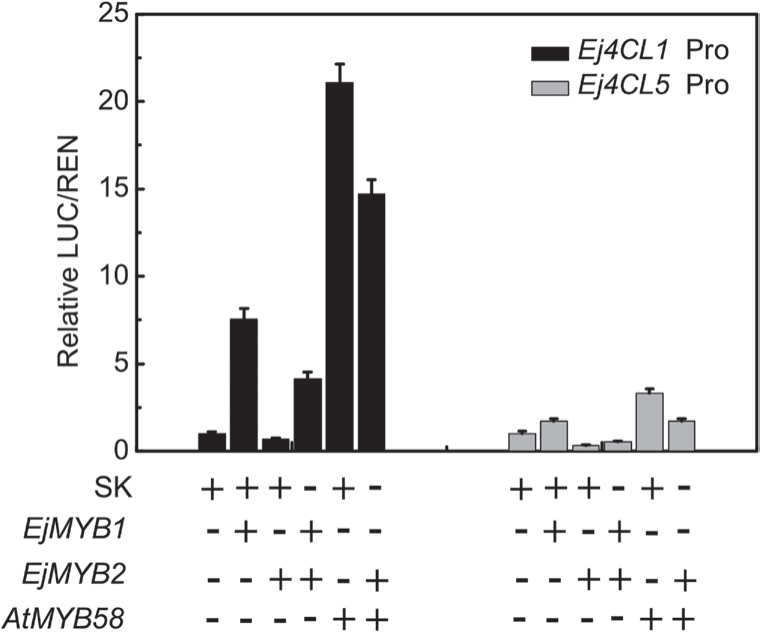
*In vivo* analysis of the repression effects of *EjMYB2* on the basis of activation of *EjMYB1* and *AtMYB58*. The ratio of LUC/REN of the empty vector (SK) plus promoter was used as a calibrator (set as 1). The symbols + and – indicate the presence and absence of the *Agrobacterium* carrying the constructs, respectively. Error bars indicate SE from at least five replicates.

A further combination experiment was conducted to show the repressor function of *EjMYB2*. *EjMYB2* strongly inhibited the stimulatory action of *EjMYB1* and *AtMYB58* on the promoters of *Ej4CL1* and *Ej4CL5* (Fig. 7).

**Fig. 8.**
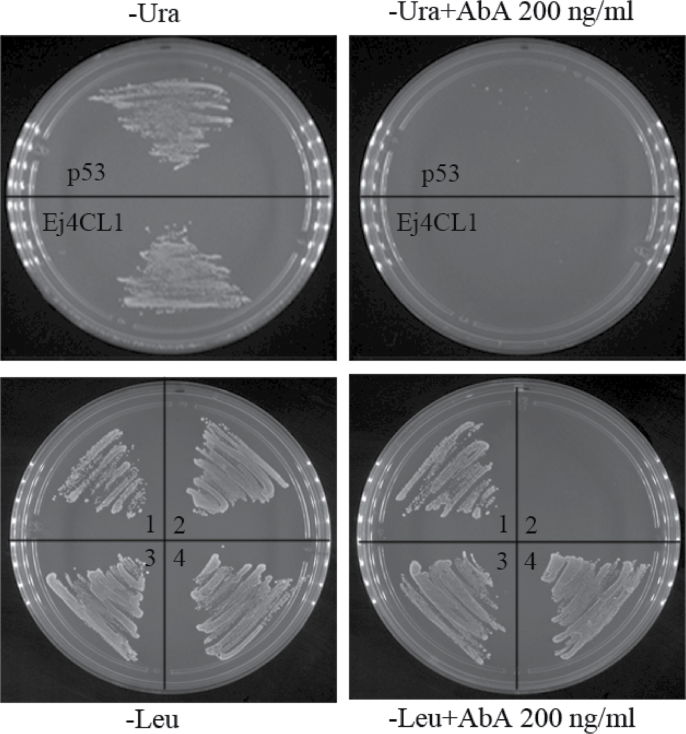
Yeast one-hybrid analysis. Numbers represent: 1, Y1HGold[p53/AbAi]+p53; 2, Y1HGold[Ej4CL1/AbAi]+p53; 3, Y1HGold[Ej4CL1/AbAi]+AD-EjMYB1; 4, Y1HGold[Ej4CL1/AbAi]+AD-EjMYB2.

To confirm whether *EjMYB2* influenced the promoter through *EjMYB1* or interacted with the target promoters directly, a yeast one-hybrid system was used. Linearized pEj4CL1-AbAi and pEj4CL5-AbAi were transformed into Y1HGold and grown on SD/–Ura medium with AbA from 0 to 1000ng ml^–1^. The promoter of *Ej4CL5* was observed to have strong activation by endogenous factors in Y1HGold (data not shown), while Y1HGold[Ej4CL1/AbAi] was suppressed by 200ng ml^–1^ of AbA (Fig. 8). The promoter of *Ej4CL1* was therefore chosen for further interaction experiments. The interaction test indicated that both *EjMYB1* and *EjMYB2* could interact directly with the promoter of *Ej4CL1* (Fig. 8).

### Transient expression of *EjMYB* genes and their roles in lignin biosynthesis

**Fig. 9.**
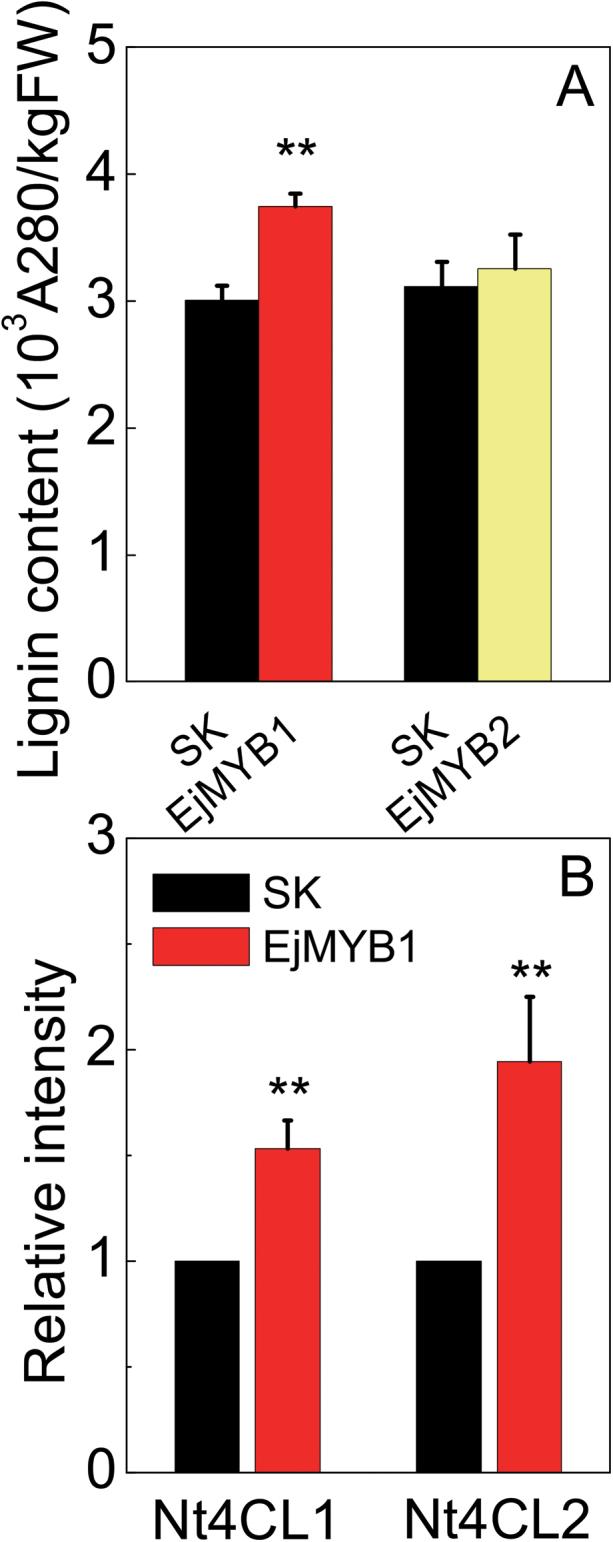
Transient overexpression of *EjMYB1* in *N. tabacum* leaves. (A) *EjMYB* genes driven by the CaMV 35S promoter. SK represents empty vector. (B) Lignin biosynthesis and the expression of *Nt4CL1* and *Nt4CL2*. Error bars indicate SE from eight (for lignin content) and three (for gene expression) biological replicates (**P <0.01).

Loquat is a perennial fruit, and a stable transformation system has not yet been reported. Thus, an ectopic transient overexpression system was chosen for *EjMYB* functional analysis. *EjMYB1* and *EjMYB2*, driven by the CaMV 35S promoter in the pGreen II 0029 62-SK vector, were introduced into *N. tabacum* leaves using *Agrobacterium*. Transient overexpression of *EjMYB1* significantly (*P*<0.01) induced the lignin content in tobacco leaves, while *EjMYB2* transient overexpression alone failed to suppress lignin biosynthesis (Fig. 9A). Nevertheless, a combination of *EjMYB2* and *EjMYB1* removed the stimulatory effect of *EjMYB1* on lignin content (data not shown).

Transcript analyses were performed on the lignin biosynthesis genes in *N. tabacum* leaves. In *EjMYB1*-overexpressing leaf blades, the endogenous tobacco lignin biosynthetic genes *Nt4CL1* and *Nt4CL2* were upregulated, compared with the blades infiltrated only with *Agrobacterium* carrying the empty vector (SK) (Fig. 9B).

## Discussion

### Chilling injury/lignification of loquat fruit and its regulation

Lignification in fruit is a rather unusual and not very common process, with most ripening fruit undergoing softening rather than hardening. However, it has been well characterized in loquat fruit (e.g. Cai *et al*., 2006*a*, *b*; Wang *et al.*, 2010) and has consequences on fruit quality. It is also unusual in being stimulated by low temperature, with this stimulation being modified by treatments that reduce low-temperature injury. These include LTC, which induces an element of tolerance to more damaging low temperatures (Cai *et al.*, 2006*b*), and heat treatments that appear to have a similar effect (Rui *et al.*, 2010). Our data in the present study, with both HT and LTC treatment, confirmed these results, effectively reducing loquat lignification after cold storage. This scenario of fruit ripening, lignification, and a low-temperature response provides an interesting model to look at the regulation of lignin biosynthesis, which may have relevance in other plant tissues and organs.

### Correlation of *EjMYB* and loquat chilling injury and fruit flesh lignification

It is commonly recognized that *MYB* genes play a regulatory role in the lignin biosynthesis pathway. As described in the Introduction, at least nine *Arabidopsis MYB* genes have been characterized as lignin related, where they have been shown to positively or negatively regulate lignin biosynthesis in various tissues of *Arabidopsis* plants. For instance, overexpression of *AtMYB85* increased lignin content in stems (Zhong *et al.*, 2008), and overexpression of *AtMYB58* and *AtMYB63* specifically activated lignin biosynthesis genes and concomitant ectopic deposition of lignin in cells that are normally unlignified (Zhou *et al.*, 2009). Similar results have also been observed and confirmed in other plants.

With this knowledge of MYB-based activation and repression, we isolated two loquat *MYB* genes, *EjMYB1* as an activator-type transcription factor, and *EjMYB2* as a repressor containing an EAR domain, according to *AtMYB58* (an activator; Zhou *et al.*, 2009) and *AtMYB4* (a repressor; Jin *et al.*, 2000). The expression of both genes was highly associated with flesh lignification. There was a consistent pattern associating low-temperature (0 °C) induction of lignification and a very strong, transient elevation in *EjMYB1* expression (Fig. 4). The two treatments, HT and LTC, which reduced initial lignification rates, also strongly inhibited this increase in *EjMYB1* expression. In contrast, the repressor-type *EjMYB2* was suppressed in lignified loquat flesh, and while HT had no obvious effect, the conditioning treatment maintained longer-term *EjMYB2* levels. Such changes suggest that *EjMYB1* and *EjMYB2* may act as regulators in fleshy fruit lignification, with *EjMYB1* particularly sensitive to temperature modulation (both LTC and HT), while *EjMYB2* specifically responded to LTC but not to HT.

The capacity for *EjMYB* genes to regulate lignin synthesis was further confirmed by transient overexpression of *EjMYB1*, which resulted in lignin accumulation, while *EjMYB2* reduced this induction by *EjMYB1*. The tobacco *Nt4CL1* and *Nt4CL2* genes were also upregulated by *EjMYB1*.

### Interaction between *EjMYB*s and promoters of genes from the phenylpropanoid pathway

Results from other plant species have indicated that the entire phenylpropanoid pathway is involved in lignin biosynthesis, e.g. quadruple mutants of *pal1–4* only generated lignin at around 20–25% of that in wild-type plants (Huang *et al.*, 2010) and antisense *4CL* transgenic poplars had around 40% lignin content compared with wild-type plants (Voelker *et al.*, 2011), and similar results have been observed with other enzymes and genes in the pathway. When promoters from *Arabidopsis* lignin genes were tested against *AtMYB58* and its loquat homologue *EjMYB1*, eight promoters (*AtPAL1*, *AtPAL2*, *AtC4H*, *At4CL1*, *At4CL2*, *AtHCT*, *AtCCoAOMT1*, and *AtCCR1*) were inducible, with *AtPAL3*, *AtF5H1*, and *AtCAD6* showing no response. The same eight were repressed by *EjMYB2*. These results support previous findings that MYB genes can be active in transcriptional regulation across the entire phenylpropanoid pathway. This has been shown through structural gene expression analysis in transgenic plants, such as *Eucalyptus EgMYB2* (Goicoechea *et al.*, 2005), *Arabidopsis MYB58* and *MYB63* (Zhou *et al.*, 2009), *Panicum virgatum PvMYB4* (Shen *et al.*, 2012), and *Gossypium hirsutum GhMYB24* (Li *et al.*, 2013).

In other perennial fruit, grape *VvMYB5a* overexpression has been shown to alter lignin metabolism in tobacco (Deluc *et al.*, 2006). However, the interaction of fruit lignin-associated MYB transcription factors and promoters of endogenous genes remains unknown. Due to the lack of genome information, a combined strategy, consisting of RNA sequencing and genome walking, was used to isolate promoters of lignin biosynthesis-related genes. Using a dual luciferase system, *EjMYB1* stimulated promoter activities of *EjPAL1*, *Ej4CL1*, and *Ej4CL5*, while *EjMYB2* inhibited the induction of *EjMYB1* on the target promoters. We then focused on 4CL, which is one of the key enzymes participating in the early stages of the monolignol biosynthesis pathway. The *4CL* gene family can be classified into two major classes (Ehlting *et al.*, 1999; Cukovica *et al.*, 2001). Members of the class I group, such as *At4CL1*, *At4CL2* (Ehlting *et al.*, 1999), *Ptr4CL1* (Hu *et al.*, 1998), *Pt4CL1*(Wagner *et al.*, 2009), and *Pv4CL1* (Xu *et al.*, 2011), are involved in the monolignol biosynthesis pathway and the members of class II group are involved in ﬂavonoid and other non-lignin pathways. In our study, *Ej4CL1* and *Ej4CL5* belonged to the class I group (Supplementary Fig. S1) and were induced by *EjMYB1*. Other promoters of key genes, such as *EjCAD1–3* did not give positive results and are missing AC elements.

As AC elements are very important for MYB transcription factor recognition (Zhou *et al.*, 2009; Romano *et al.*, 2012; Shen *et al.*, 2012), a deletion experiment was performed and the results indicated that interaction of loquat *EjMYB1* with the *Ej4CL1* promoter also required AC elements. *EjMYB1* activation was progressively reduced with fewer AC elements (Fig. 6c). This was partially confirmed in the yeast one-hybrid experiments where *EjMYB1* and *EjMYB2* interacted with the promoter directly. These results are highly indicative of *EjMYB* genes being involved in transcriptional regulation of lignin biosynthesis in loquat fruit, particularly through manipulating *Ej4CL* genes.

In conclusion, the new information in this study is that *MYB* genes are involved in chilling injury and flesh lignification, shown by utilizing loquat fruit. The particular targeting of *Ej4CL1* raises issues of detecting other regulators in different parts of the pathway responding to different stimulation. In addition, as well as published work on other plant species, we have shown that *EjMYB1* and *EjMYB2* are chilling responsive and modulated by postharvest temperature treatments, such as LTC and HT, in different ways. The results also indicate that *EjMYB* genes have transcriptional regulatory roles that have close homology to those of *Arabidopsis* genes, and a commonality in recognizing and competitively binding to AC elements in lignin biosynthetic gene promoters. Thus, the present research helps to expand the functional categories and regulatory mechanisms of MYB transcription factors.

## Supplementary data

Supplementary data are available at *JXB* online.

Supplementary Fig. S1. Phylogenetic tree of Ej4CL.

Supplementary Fig. S2. Sequences of promoters of lignin biosynthesis-related genes from loquat fruit.

Supplementary Table S1. Primers for *EjMYB1* and *EjMYB2* isolation.

Supplementary Table S2. Primers for *EjMYB1* and *EjMYB2* UTR region isolation.

Supplementary Table S3. Primers used for isolation on promoters of lignin biosynthesis genes from *Arabidopsis*.

Supplementary Table S4. Primers for isolation of promoters of lignin biosynthesis genes from loquat using genome-walking technology.

Supplementary Table S5. Primers for *Ej4CL1* promoter deletion experiment.

Supplementary Table S6. Primers used in subcellular localization analysis.

Supplementary Table S7. Primers for real-time PCR.

Supplementary Table S8. Primers used in yeast one-hybrid experiments.

Supplementary Data

## Abbreviations:

4CL: 4-coumarate:CoA ligase
CAD: cinnamyl alcohol dehydrogenase
CaMV: cauliflower mosaic virus
CCR: cinnamoyl-CoA reductase
C3H: *p*-coumarate 3-hydroxylase
C4H: cinnamate 4-hydroxylase
EAR: ERF-associated amphiphilic repression domain
HT: heat treatment
LTC: low-temperature conditioning
LYQ: Luoyangqing
NLS: nuclear localization signal
PAL: phenylalanine ammonia lyase
POD: peroxidase
TAIR: The Arabidopsis Information Resource.
