# Supplementary material for: Activator- and repressor-type MYB transcription factors are involved in chilling injury induced flesh lignification in loquat via their interactions with the phenylpropanoid pathway
Source: J Exp Bot. 2014 May 24;65(15):4349–59. doi: 10.1093/jxb/eru208 (PMC4112638; doi:10.1093/jxb/eru208)
Supplement: Supplementary Data [file supp_65_15_4349__index.html]

Activator- and repressor-type MYB transcription factors are involved in chilling injury induced flesh lignification in loquat via their interactions with the phenylpropanoid pathway — Activator- and repressor-type MYB transcription factors are involved in chilling injury induced flesh lignification in loquat via their interactions with the phenylpropanoid pathway — Supplementary Data 

# Activator- and repressor-type MYB transcription factors are involved in chilling injury induced flesh lignification in loquat via their interactions with the phenylpropanoid pathway

## Supplementary Data

Data files

**Files in this Data Supplement:**

- Supplementary Data - Supplementary Data
