# Supplementary material for: Activator- and repressor-type MYB transcription factors are involved in chilling injury induced flesh lignification in loquat via their interactions with the phenylpropanoid pathway
Source: J Exp Bot. 2014 May 24;65(15):4349–59. doi: 10.1093/jxb/eru208 (PMC4112638; doi:10.1093/jxb/eru208)
Supplement: Supplementary Data [file supp_eru208_jexbot120840_file001.pdf]

Activator- and repressor-type MYB transcription factors are involved in chilling injury induced flesh lignification in loquat via their interactions with the phenylpropanoid pathway

Qian Xu[1,#], Xue-ren Yin[1,#], Jiao-ke Zeng[1], Hang Ge[1], Min Song[1], Chang-jie Xu[1], Xian Li[1], Ian B. Ferguson[1,2], Kun-song Chen[1,*]

Figure S1

Phylogenetic tree of Ej4CL and other 4CL members from some plants. Ej4CL1 and Ej4CL5 are classified in the class I group which is devoted to the monolignol biosynthesis pathway.

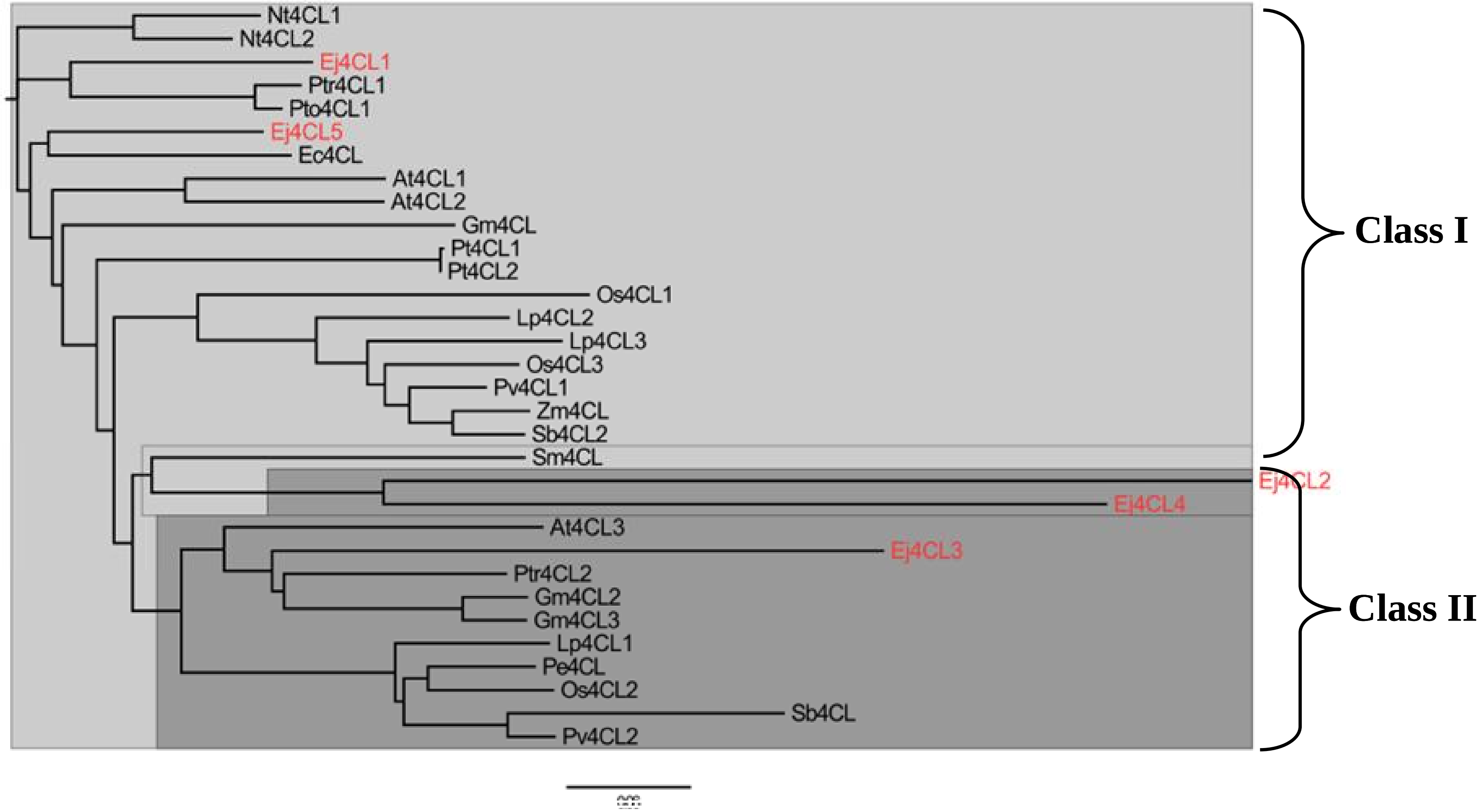

Figure S2

Promoter sequences of EjPAL1, Ej4CL1, Ej4CL2, Ej4CL3, Ej4CL4, Ej4CL5, EjCAD1, EjCAD2, EjCAD3.

>EjPAL1 promoter
CTGGTATCCAAAATCTTACTACGTTAATGCTGCTAAACTGTAAATCTTGATTATAATTTACATTTACATCATCATAAACTACGCATGTTGTTCTAAAACACACTTGTACGCCATGTGTCATCACTAGAA
TGGTGTAGTTTACATATTCCAACAAAAATTTACATCATCAAGAATTTACATTTACATTTTTAAAAGTTTAACTGACATAACATGTTACATTTGAATTTAATTCAAGATAATCTCTTTACGGTTTGTACAC
TTTCTTTACGTTCAATTAAAACCGCTATCGTTTCACCTTAAAAAAAAACACCAACACCTTACATCATACGACAATGCAACGGCACCTTGCACGAAGAATTTTATTTGTCATATGAACAATTGTTTTTG
CAGAGAAATAGGCTGACGTGCTGCTGTCGTGCCTTAGTTTCCTTCAGAGAGCCTGCTTGCTCACCAACCCCCGCCCCCCTCTTCCCTTCCCATTCGGTTATCATTTGACAAGTAAAAGCCCGCGTT
TTTGAACCACACCTAAACTACACATGTTGTTCTCAAACACACATGTACGCCACGTGTCGCCACGTGAACGGTCCTAGATCATGAAGATCCAAGGGCCACCTGCAGTTTTCAACTAACACTTCGGC
CTACCCACCTAGCATTCGATGTGGGACGTCGCCTATATAAACGAGACAATTATGGGTGAACCAACTCAGGAAAATACTCGTAAATTTTCCTTTTTACCCCAAATTTAACTCAGAGCTAGCACCACCT
CTTTCCATTCCCTTCGTTTCCCAATTCCCATTCCCTTATTGTTTCTCTTCGACCAGGTTTTTACCCCACTGAGTTTTTCCCGGCAAGGTTTTTCTCGGCTCCATTCATTCTCATTGTTTCCTATCCCCAT
TTTGGTATTGTTTGTGATCTTGGAAGCAAATG

>Ej4CL1 promoter
CTGGTAAAAATGCGGTTTGGAAACTTTGACATAAATGGTATAAATCTAACCGCACAAAGCAATTCATTAAATATTCAACCACGGCAAGTAATACAGTAGAATTTTGGTGGAATTTCCAAGTCAGGTA
TTTGGTAGAATTGTGAAAATCACTTTCACCAAGAAGTTCTGAGACGCGCATCTCCTAAATCATGTCGTTGTGATAGTAGTAGTAGGTAAAGGGCAGTATGGTCTTCGCAGACGCCAATTCCTTTGT
CAGCCAGTGGTTGGTGTAAAGCCTCTGCATTTAGAAAATGTCCTGACCCACCGCCACCATCCAAACCCACTTTCACCAACCAAACCCCTTGCACTCGTCCCTTCCGCCTCCGGTCACCTATCACC
ACCAACCCCGAATGTGTCTTATCAAATTTCCAATCTTTCACCATCACGGTTTCACCCCCAAACAATTCCATAATCCCATTTCCCTCCCCCCCACCAACCACACCTCCAGTTGTATATGAAGAACCCC
TATTGACCTCCACTCTTCCCTCAAGCACCAATTCAGCTCAAGATAAACCAACAATTTTGGTGTCTTTTTTCTCATTGGATAATCCCATTTTTCCAAGAGATG

>Ej4CL2 promoter
CTGGTATTTATTGATATAAAATAAAACGTACAAAACGGAGGATAGTGTGCTTAAATGGGCCTTGGTAAAAACCTTGTCGGGATTTTCAACATGGTCGAAGGGAAAAAGAGCGTCGCGCATGAACA
ACTAACTTTACCTATCCTCACACCAAGAGAGAAAACAAGAAAAATTATAAAGAGTTGTCTTCCATAAGAGTATCCATCAATAGCTCAGGAGCAGCCCCCCTCCATTGAAATTCATCAATCCTGCCA
ATGCCAGATCGAACAAGTCGATGGAAGCATTGGTTTATACATTCATTTTAGTCTCAACTCTAGGAATAATTTTTTTTGCCATCTTTTTTCGAGAATCATCTAAAGTTCCAACTAAAAGGTGAAATGAC
TTGTCATTATCTCAATTGAAGTATAGACTTCAACTGGTCCTTGGGTTCCTCTAATGGATCTCTTAGTTATTGAGAGGGTTGCCCAAAAGCAGTATATAAGGTGTACCCAGGAAAACATACAAGTAAA
CCAGATAAAGAAATGGCAACTAGGGTTATTGTTTCCAATATTACATAGTTTTAAGACAATATTACTTTTTTATGATACAACAATATATTTACATTAAGAATGTGAAAAGATAAATTAGTTTCGAACCTA
CCGCACGAAATTCTAACTTATTAATGAAGAACTAAGAAAATTTTACTAAATTACAACCCCGAAATCTAATCCTTCAAAAATGAGAAAAATATAGTTATGAACCTATCCTTGTCGCAATTCATTACTAT
ATGACCACTTGCCTCAAAAGTCTTCACATGTAGATGTAGTTATATTTTTGTCTGAAATTAACATGCAATAGCGTTGAACTTTGCTGATTTATTTTCTATTTGCCACTGCACAAATCCCATCAGTTTCGC
CGCCCGTCCGCTATTTTACTTGTCACTTCTGAAAGCAAAAGATATTAAAAAGAAAAATTATAAAAAAATTAACAAAAATTAAAAAAAGAAGTAAAAACCCATCACCCGTATCAGAAGAGAAGAAA
AGGCAAAAATTGCGGACCAAGCATGGCGTTCCATTGCCACCCTCCCGCCGTGAAGTACTGAATGAGTGACTGAGTCTTGCGATCCGACGCGAAATACTTGCCGCTTCTCCATGTCTCTTTTGTCCT
GTCGCAAATAGTCGTATATCGACACGGAGCAAGAGTTTACACCGAGTTTGTGCCGTGTTTAGCTTGAAGGAAACAAATCTAGCAATCCACTTGAGTTTAAGTTTTACCAACCAGACCGGTAAACA
TTTCCTAACAGGATCACAGATTCACAGCACCTCCTTAGCAAATCATAAGACAACAACTTTTTGCCGTAAAAAAAAGAAACAATCAATACACCTCGATACGTGTGGTACTCCATCACGTATCACATAT
CACCTCATCCAAATCCCAGAAACCCGAAATTCCAGCTTACCCAGTTGGGCCTCTCCACCGCAATCTTCAATG

>Ej4CL3 promoter
CTGTCGACAGTATGAAGGCTTGGTTGGTTGATGATTGAACGTGTGGTAAGGCATGCTTCTCCTCCTAACCCTTCTACCCCTCAACTTCAACCCCATCTGCCAAATACTCAACACAACCCTCCCCTC
CTCCTCTTCTCTCCCTTTCTCTAACATACACAGAGCAACCCTTGTTCCTACCTACCTCCCTCCTCACCCGATAGATTTTCAGTATAACTGATACACAAGATGGTACATCACATGTCACTATACAAATG
GTAGGACATGTGTGCTAAACTTAAAAAATAAAATTTCTCACTACTCACATGTATCTTGTATTCCGTCACAGTTAAAAATTTCTCCTCGTCAACCCGTCTGGCCTCTCCTCCTCGTATTATTATATC
TTACAAACTCCACTTTTTTCTGATAAACAACAACGGCGCACACGTAGCTGCTCTGTCTCACACACACACACACAGAGCAACCCTTGTTCCTACCTACCTCCTCCTTCTTACCCCATCTCCTTGTATT
TATTACTTCTCACAAACTCCACTGTTTTTGTGACCAACAACAACAACGCGCACGTAGCTACTCTCTCTCTTCTCTCTGATCATCATTTGATTCATTTCTGGTAGTCTGCTATATTATATCAAGCCTCCC
TCTTTCTCAATTCTTATTTTTCTGTATATTTCAATCTGTTTATTGGTCGATATATCACACACAAAGAAGCAACAGTCACCATG

>Ej4CL4 promoter
AAATAGAGGGTGTTGAAGTCAACTAAGAAAAGAGTAAGCAGAGAGTAGCAATCTCCTGTTCATATTAAAAATAAAATTACTTTATTATCTTAAACGTTTGTCATCATCAAAATTAATAAAAATTTGA
ATAAAGAATTCTTAAAATCTAACACATGGATAAATTGGCTGATTATAAAATTTGAAGGGAGAATCCAAATTAAAAATTAGAAGAGAAATTAGTAGCTTCTTATAAATTTAGAAAAACTTTTATGTGTT
ACCCTTATTTTGTTTAACAAATAATATTATCTACACTAATGAGAGGGTTTATACTAGCAATAATATGATTCAAATTCGTTTTTGGTAAGAATCGAATCTAAAACCTTTCATTTCACTTACAAATGAAGA
GAAATATCCCTAAATTGTAGTATTAAGTGACATCTCTTTCACATTTATGGTTTTTTTTTTCAATTTATTTGACATTCAAAAGTAAAAACAATGCGTAAAAATAGAAGAAAAAAATGTGTATAACACCA
TTCTAAAACGGACATGATGAACCACTAATTTCAGTGCTCTTAAAGAAAACTGACTTAGTTTCCACAAAAAAATAATAATAAGAAAAAAAAAAAGAAAAAAGAAAACTGACTTTTTATAGAACACG
ACAACTCATAATATATTACTCCTCCCGAAGAGAAGCACTTTCAAGTGAAACCTTCAACTTTCTGATCAAAAAAATAGGGAAACCTTCAACAATG

>Ej4CL5 promoter
CTGGTAAATTCCAAAAAAGATTAATCAACTTTTTCTACGGCAAAAAAACAAATTAAAAAAAAAAAACTGGAAATACAATTAAGGAAAATACAAAAAAAGAAAAAGAAAAAGAAAATGTGAATATT
TAAGCCAGCCAGCCAGCCATATCTGAGCCAGCAGCCAGTTTTAAGCGAGTGAGCGTGACAACATTACGACGGCACTTGGGCGTGGATTTTTATGTCCATGACTAAGCACTTGTAGTAGGTGAATG
GTGTCGCTTACTAGAGTCACCAACCAAGGGACGAGTAATGACTCTGCGTTAGGTGAAGCGTCATACGTGGCAGCCAACTGGTTGGTTTTGGGCTAAAGTGTGCCGCATGTAACCAAATCCATTTC
GCTTCCCGAAACCACCAAACATCCCTCACCTATTACATAACGCCACGTGTACTAGAGATGCGAAGCTGACTTAATAAAGAGAAATAAATTGTGTACGTGGAATGGACTGATTGGTCTGCTACTTCG
GTGACGGCCATGTGGATGAAAGAGTTGGTGACAAATAAATTAATACCAACCAAGTCCGTTTCCCCCACCACACTTGCTTGTATATTTACTTCAACCAAAACCCAAACCAAAAACCAGGGAGAAGG
TCGTCTGAAAGAAACCAACCCAATCCCTACTACCAATTTTCTCTCACATTTTCCCTCACTTTCTCACCATCCAAACACATATCCAATG

>EjCAD1 promoter
ATCCCTAGATACACTAGGATTTACACAATCACATTCCTAATCTAACATCCAATTAGGTTGAAAAAATATAAATATTTAGACTGTGTTTTAGAAGAGCCTAGATTGTGATGATGAATCTTGTGAACGGG
CCTTGGCGATGTACTTATAACGTGTTGTAGTAGGCCTATTTACTGAGAGAAGAAAAGAGGGAGATCGGCCAGAGAGAAGAGACAGGAGAGAAGGGCGGCCGAATAGAAGAGAAGGTCTAAAGG
AATTCTATGTGTTATTTCTCATGCTCTGTGCCTTTTTTATACTATTAAGGAAGAGATAAATTTACTCTCTAATTAATACAAGTTGTACAAGAAGAGATCATCGAGATCTTATAGGAGTCCTATACGTAAT
CTTATACTCCAAATAAAATTAGGAATGCAGCATAAATTAGTTTTTTTTGTTTTGACACTTTTTTTTTATTGATTTTTTAATTTCAAACAAACGATATTATCTATACTAAGGGATAAATGATGGATTTAGCT
TTACAATATACTAACAATAATGTTGTTCAAATTTACTTTTGATGATAGTCAAAGTTAATACCTATCATTTACAAAAGAAAAAAACACTACAAGACTATACTAGTAAGTGGCGTGGTTGGAGATGTAGT
AATAGACTAGATCATCAGCTACGTGCCTACGAATGCCAATACGCAACAATTCCGTTCTGAAACAGACAAAAAGCATTTTACGTAAAAGGAGCTTCGAAACTTCAAAACGTAAGATCTGCAGAGTG
AACACACACCCACATATAGAGAGAGAGAGAGAGAGAGAGAGAGAGAGAGAGAGAGAGAGAGAGAGAGAGAGATG

>EjCAD2 promoter
CTGGTCCTTTTGGGAGCTCACTAGCTTCGGGTTCCGTAGAAACTCCGAAGTTAAGCGAGTAGCGCGTGAGAGCATTCCTATGATGTGTCCACTGGGAAGTTCTTGTGTGAGTTCCTAGAAACAAA
ACCGTGAGAACGTGGTCGGGGCCGAAAGCGGACAATATTGTGCTACGGTGGTGGAGCGGGCCAGGGATGTGGTGGGGGCCCAGGCCGGGATGTGACATTCGGGATCATCTTAGGCTCCAAAAA
CGCAATTCAAGCCCAAAACGTCACTTATTCATCCATTTGACCATGTTTTTCTCCAGAAGAAGTCGAAAGTCCTATAATGAAAATACAATTCGAAGTATCAACATTCTTCCAAAATATTAACCAGAAT
ACACTAAGAATAGGGTTAAATATATAATACGAAATGTACTCATCAGAAAGTGAGGAAGAGAGAGAGTGAGGTCTGAGAGAAAGCTGCCACATGGCATGCAATGGGATGGGAGATCCGGGGGAAC
CAAAATCAAGGGTGGGCCCCTTGGGCATATCATTTAACACCAAAACAATATTTTAAACAAATATCCCACAACCCAAATGAATTTACCAAAAATATCCTTCTGTTTCGTAAAATCCGGGACAAGTTGT
CACAAGGAATCTTTGAAGGGAAGCTTTTGGGTAGCATACATAGATTTTTATTATAAGAAAAAAGTAACCTTAATCCCAAGTTAAACAGCGAAATCACTTCATAATCCCTAAATACTGGAGAAGGAA
TTTTTGAAAGGGAAGCTCACCATGAGCACAGACACCTTTGTAGGCTTCCAACAAAAGTCCCAAAAAACGAAAAACCAGGGTCTTCTAAAATTGAAAACAAAACCAAATTAAACCACCACACGT
AGAATCGCAACCACAAACAAAATCCCCAACACAATCCCAAATGGAAGGAAAGGGAAGTGGAAGAAGAAGAGTGGCGACAGAGAGGGAAAGATACAATCCGTTGACCCCACCCTCTCTTTGTA
GCCAACACACTCATTCTCTCAACCCTATCCTCAAACCGTGCACCTAATCAACTCTCTTAGTCATTTATCGTACATCGTACAGTCAGAAATCATTTGATTTTTTATTTAAAATTAAACACAAATATTATC
TAACGAAAACTAACCGTACAATGTACGATGAACGGCTAAAATGTAGAGATCCTTAGGATCTCTAAAATATGGATCCGGAGATGATCCCCTTCCCCGAGCTGAAGAGCACAAGTTGGCAACATTTAC
CTTTAAATACCAGATGACTTTTCACCATGAAAAACACAATTCTGAACCAAATTAAGCTTATTAATTCTTAGCTAGAGAGAGATATG

>EjCAD3 promoter
CAAAAGTTTGGGATATATATTGTGTGTGTGTGCGCGTGCATACATACATACATACATATATATATATATATCTGATATAAATTATAAATTATTTAGATAATGTTTTGTCTTTTTCCTAGGCAAGCATCATTCAC
ATATGTATGTTCTTATATAAGAAATAGTATATTAGTCAATAATTATTTACATTTGATATAGTAAATTTAATTAATTCATAAAATATATAATAAATTTACCTAAATTCGTTTAGATCTCACCTAGCCGCCTAGG
CGCTAGGCGCTAACCTGCCACCCGACTAGAGCGTAGCGTTTTGAATCTTGCTTTCCATTGATAATGGTATCTACTTCCTTCCTTATGGGTCATCTATGGATAGTGATGGGTTCAGGTCGAGAAGATTT
TCATTGTGACGGGAATATGAATGGTACATTACGTGTTTTTATGTAAGTAATGAAAAATTTTATTTTTTAAGTTATTAACTTTTTTAGAGTAACACGTAGTGTATTTTCTCATATGCTGGTTACACTGAAA
AATATCTTTTTCAGGTTAGTCAGGGCAGATTGAATTAGTATAAATTTTGGTGGGAAGGAGTAAGATTCTCTTTCCTCTTATTTTCATTTTCTTCCTTTCCTTTATGTCACACTTTGTTTTTTGTCTTATTA
TCTTTATAAAATATTAATATATGATATTAGCATAATTTAACTGTAATTATTCAAGTAAAATGAGAGAGAAGAGAAGAGAGAAAGAaTAGAAAATGAAAGaATCCTCGTCCCCAAGTAAACCTGTTGAAAG
GTACCCAGGTACCGAAGTCATCACTGCGTACGTTAATGGAGGTGATCACGCATGCTCTTGCGTCACCCAAAAACCTGCACGTAAGTCTATAAATGGTAAAATAGCATCTCTCGTGACAACCGAAA
CCCAAAAACTTAAACTGTTCACCAAGATAGAAAAAACCCAAAAACCTAGGGCAAAACGACTATTTTCTCAACAATATCTTTAATTTCTTCCCAATTTCTGGGGAGCCCTCTGCATATTTACAACAA
ATTATTAAGGGTCCCAATTATTAAGCATG

Table S1 Primers for *EjMYB1* and *EjMYB2* isolation

Gene	Forward primer (5′ to 3′)	Reverse primer (5′ to 3′)
EjMYB1	ATGGGGAGAGCTCCTTGCTGTG	TCATACCAAAATTTCCATGCCAT
EjMYB2	ATGGGAAGATCTCCTTGCTGT	TCATTTCATCTCCAAGCTTCTG

Table S2 Primers for *EjMYB1* and *EjMYB2* UTR region isolation.

Gene	Gene-specific primer (5′ to 3′)	Nest Gene-specific primer (5′ to 3′)
EjMYB1 (5′RACE)	TATTTCGTTATCGGTGCGTCCTGGTA	AATATCCGGCCTCAAATAGTTTGTCC
EjMYB2(5′RACE)	AGGAGGCTATGGAGTTTGATGATAAGC	CGCTTGAGATCAGGTCTAAGGTAGTTG
EjMYB1(3′RACE)	AACCTTCCCCGATGTTGATGACAGC	CGGAGTTTCCCATCACCAAAAACG
EjMYB2(3′RACE)	CGCAAGCATTCAACAAACCCAGTTC	TGCAGTTTGGGGATTCAAGATGCAA

Table S3 Primers used for isolation on promoters of lignin biosynthesis genes from *Arabidopsis*

Gene	Forward primer (5′ to 3′)	Reverse primer (5′ to 3′)
AtPAL1	CAATTAACCAACACACCACTTCTTC	TCTCCATGGAGACTTTTGATCTTAGT
AtPAL2	AACGGTGTGTGTTTCTTTACTTAGC	GATCCATGGGTTTTCAAGAAGT
AtPAL3	CCCAACTACAAATTGTTGGACTACT	CTCCATGGTCACAAAATCTGACGG
AtC4H	GTGGTAATGGTAGGTGTGTCTCTCT	GTCCATGGTAGTTTGTGTATCCGCA
At4CL1	TCTGAGTTATGCTTTCGTGAACA	GCCATGGTGTGCATTTACAATAGT
At4CL2	AGCTACAGCTTCCATTGATTCAG	TCGCCATGGATCAGAAGTTAATATC
AtHCT	AAGCTAACTGAACAGAGACGACATT	ATTTCCATGGTTTTAAGAAAGATCA
AtCCoAOMT1	AACAGTTGTGTACACGTAGCTCTCA	CGCCATGGCTCTCTCTTTCTCTCT
AtCCR1	GTTCTCCAGAAGTCAGGTATGAAAG	ACTGCCATGGTTCCCGGTGGACG
AtF5H	GTGAAACGAGCGTGACTTAATATG	GACTCCATGGTGAGTGTTTTTTTGG

Table S4 Primers for isolation on promoters of lignin biosynthesis genes from loquat using genome-walking technology

Gene	Gene-specific primer (5′ to 3′)	Nested gene-specific primer (5′ to 3′)
Ej4CL1(P1)	AGTAGGAGTGGAGAGGGAGGTGTTTT	TGGGGTAGTTTCTATAGCCATGGCTTGGA
Ej4CL2	AGGAGGAGGAGTTTCTGAAGAGGAAG	ACCCAGATTTCTCCATGGAAGATTGC
Ej4CL3	TTAATCAGAGGAGGCATGAGATTGGT	AGACCATGGTGACTGTTGCTTCTTTG
Ej4CL4	GATTGGAGGAGGGAGAGGGTGAAT	GATTCCTCCGCCATGGTTGAAGGT
Ej4CL5	GGAGATGTTTTCGAAGCAGTAGGTGT	CTCCATGGGATATGTGTTTGGATGGT

Table S5

Primers for Ej4CL1 promoter deletion experiment

Gene	Forward primer (5′ to 3′)	Reverse primer (5′ to 3′)
Ej4CL1(P2)	TGCATTTAGAAAATGTCCTGACC	TGGGGTAGTTTCTATAGCCATGGCTTGGA
Ej4CL1(P3)	GCACTCGTCCCTTCCGCCTCCGGT	TGGGGTAGTTTCTATAGCCATGGCTTGGA
Ej4CL1(P4)	AAATTTCCAATCTTTCACCATCA	TGGGGTAGTTTCTATAGCCATGGCTTGGA

Table S6

Primers used in Subcellular localization analysis

Gene	Forward primer (5′ to 3′)	Reverse primer (5′ to 3′)
EjMYB1	GGACTCTAGAGGATCCATGGGG	CCATAAGCTTGTCGACTACCAAAA
	AGAGCTCCTTGCTGT	TTTCCATGCCATCATC
EjMYB2	GGACTCTAGAGGATCCATGGGA	CCATAAGCTTGTCGACTTTCATCT
	AGATCTCCTTGCTGT	CCAAGCTTCTGTAATC

Table S7

Primers for realtime PCR

Gene	Forward primer (5′ to 3′)	Reverse primer (5′ to 3′)
EjMYB1	TGCTGTCTTCTTAGCATTTTTCA	CCCCTTCTTCAATCCCATCT
EjMYB2	CAAGCATTCAACAAACCCAGT	TCCTCTCCCTCCACTCTTCA
EjACT	GGATTTGCTGGTGATGATGC	CCGTGCTCAATGGGATACTT
Nt4CL1	GGTTACACACTGGCGACATTGG	GGAACTTCTCCTGCTTGCTCATC
Nt4CL2	AAGGACGAGCAAGCAGGAG	AAGGATTTTGCCAGATGGAG
Actin	AATGGAACTGGAATGGTCAAGGC	TGCCAGATCTTCTCCATGTCATCCCA

Table S8

Primers used in yeast one-hybrid experiments

Gene	Forward primer (5′ to 3′)	Reverse primer (5′ to 3′)
EjMYB1	GGAGGCCAGTGGATCCATGGGGAGAGCTCCTTGCTGTGAG	CGAGCTCGATGGATCCTCATACCAAAATTTCCATGCCATC
EjMYB2	GGAGGCCAGTGGATCCATGGGAAGATCTCCTTGCTGTGAG	CGAGCTCGATGGATCCTCATTTCATCTCCAAGCTTCTGTA
Ej4CL1	AATTCGAGCTCCTGGTAAAAATGCGGTTTGGAAAC	CTCGAGGTCGACCATCTCTTGGAAAAATGGGATTAT
